# Prediction of patient choice tendency in medical decision-making based on machine learning algorithm

**DOI:** 10.3389/fpubh.2023.1087358

**Published:** 2023-02-24

**Authors:** Yuwen Lyu, Qian Xu, Zhenchao Yang, Junrong Liu

**Affiliations:** ^1^Institute of Humanities and Social Sciences, Guangzhou Medical University, Guangzhou, China; ^2^School of Health Management, Guangzhou Medical University, Guangzhou, China; ^3^The Eighth Affiliated Hospital of Guangzhou Medical University, Guangzhou, China

**Keywords:** machine learning algorithm, prediction, patient choice tendency, medical decision-making, assistance systems

## Abstract

**Objective:**

Machine learning (ML) algorithms, as an early branch of artificial intelligence technology, can effectively simulate human behavior by training on data from the training set. Machine learning algorithms were used in this study to predict patient choice tendencies in medical decision-making. Its goal was to help physicians understand patient preferences and to serve as a resource for the development of decision-making schemes in clinical treatment. As a result, physicians and patients can have better conversations at lower expenses, leading to better medical decisions.

**Method:**

Patient medical decision-making tendencies were predicted by primary survey data obtained from 248 participants at third-level grade-A hospitals in China. Specifically, 12 predictor variables were set according to the literature review, and four types of outcome variables were set based on the optimization principle of clinical diagnosis and treatment. That is, the patient's medical decision-making tendency, which is classified as treatment effect, treatment cost, treatment side effect, and treatment experience. In conjunction with the study's data characteristics, three ML classification algorithms, decision tree (DT), k-nearest neighbor (KNN), and support vector machine (SVM), were used to predict patients' medical decision-making tendency, and the performance of the three types of algorithms was compared.

**Results:**

The accuracy of the DT algorithm for predicting patients' choice tendency in medical decision making is 80% for treatment effect, 60% for treatment cost, 56% for treatment side effects, and 60% for treatment experience, followed by the KNN algorithm at 78%, 66%, 74%, 84%, and the SVM algorithm at 82%, 76%, 80%, 94%. At the same time, the comprehensive evaluation index F1-score of the DT algorithm are 0.80, 0.61, 0.58, 0.60, the KNN algorithm are 0.75, 0.65, 0.71, 0.84, and the SVM algorithm are 0.81, 0.74, 0.73, 0.94.

**Conclusion:**

Among the three ML classification algorithms, SVM has the highest accuracy and the best performance. Therefore, the prediction results have certain reference values and guiding significance for physicians to formulate clinical treatment plans. The research results are helpful to promote the development and application of a patient-centered medical decision assistance system, to resolve the conflict of interests between physicians and patients and assist them to realize scientific decision-making.

## 1. Introduction

Traditional medical models are gradually shifting from disease-centered to patient-centered as medicine progress (1, 2). The Institute for Patient-and Family-Centered Care (IPFCC), an institution specializing in patient-centered medical services in the United States, proposes that medical services should involve four elements: respect and dignity, information sharing, participation, and collaboration (3). Patient involvement in medical decision-making has recently become the focus of research. It is also a significant issue in clinical practice (4). According to a survey conducted in the United Kingdom, more patients want to be involved in their medical decision-making. Specifically, one-third of community patients and half of hospitalized patients want to participate in the determination of treatment plans (5). Schoenfeld et al. (6) discovered that when there are multiple reasonable options, most adult emergency department patients want to participate in part of the decision-making process.

Patients' participation in medical decision-making has been regarded as a sign of the quality of medical care (7), which is conducive to the establishment of a harmonious doctor–patient relationship, the formation of correct diagnosis and treatment plans, and the reduction of medical expenses. This improves patient satisfaction and treatment effect, so the concept of patient participation in decision-making is widely accepted. However, there are many barriers to patient participation in medical decision-making, such as insufficient doctor–patient communication, a lack of patient health literacy, limited diagnosis, and treatment time, and other factors that lead to patients not knowing how to participate in decision-making. As a result, communication between physicians and patients is critical. Physicians need to inform their patients about different treatment options, listen patiently to their appeals, and discuss the risks and benefits of different treatment options with them. Similarly, patients should inform physicians about their preferences in medical decision-making. These factors will influence the final decision; thus, patient preference is undeniably important in medical decision-making. According to the principle of medical decision-making optimization (8), physicians should formulate diagnosis and treatment plans with the goal of maximizing benefits while minimizing costs. To be more specific, according to the patient's condition and family situation, as well as the development level of local medical technology and objective conditions, the diagnosis and treatment measures with the least pain, the lowest cost, the best curative effect, and the highest degree of safety should be taken. According to this, patients' medical decision-making tendency was set into four aspects, namely, treatment effect, treatment cost, treatment side effect, and treatment experience.

In recent years, more researchers have attempted to predict medical problems using Machine learning (ML), deep learning, and neural network modeling methods. Researchers at the University of Pittsburgh created the first clinical decision support system in human medicine in the 1970s, with the goal of diagnosing complex internal diseases (9). Zhou et al. (10) focused on modeling and analyzing doctor–patient generated data based on an ensemble CNN-RNN framework. In order to improve patients' access to high-quality health information, medical resources, and professional guidance in a virtual healthcare setting, and therefore to promote patient participation in shared decision-making. Sun et al. (11) constructed a deep learning-based medical image and transcript data analysis model. According to the analysis results of medical big data, this can intelligently judge diseases and make effective decisions. At the same time, it can analyze the health status of patients according to the medical examination records and predict the risk of a certain disease in the future. During the COVID-19 pandemic, Pourhomayoun and Shakibi (12) extracted characteristic symptoms of COVID-19 patients that should be paid attention based on the prediction model of the ML algorithm, and the prediction accuracy of their mortality was as high as 89.98%. Specific application studies include leukemia diagnosis (13), prediction of death risk in patients with sepsis (14), formulation of drug dosage in patients with radiotherapy (15), diagnosis of allergic rhinitis (16), etc. Although algorithms are not unfamiliar to medical decision-making, the availability of large amounts of medical data makes ML increasingly applicable in this field (17), and its scope of solving decision-making problems gradually expands (18).

The “patient-centered” service concept, which emphasizes patient input into healthcare decisions, is gaining popularity. However, in the specific medical decision-making implementation process, evaluation tools, and quantitative models are urgently needed to assist physicians to make judgments. As a result, it is critical to implement ML algorithms to help physicians understand their patients' medical decision-making preferences. Due to the large number of variables involved in medical decision-making and the difficulty of data acquisition, it is a challenge to predict the choice tendencies of patients. There are, however, few studies that use the ML algorithm to predict and analyze choice tendencies in medical decision-making. The purpose of this study was to investigate the use of traditional ML algorithms in the prediction of patient choice tendencies in order to improve the quality of medical decision-making.

## 2. Materials and methods

### 2.1. Data and sample

The data for this study were obtained from 248 valid patient questionnaires collected by the project team members at third-level grade-A hospitals in Guangzhou by random sampling from September to December 2021. Before the implementation of the questionnaire, the investigators explained the study to the hospital's medical staff and patients, and all participants provided written informed consent. Ethical approvals were obtained by the Ethical Review Committee of the China Guangzhou Medical University. The questionnaire data consists of two parts. The first part includes the basic information of the patient, and the second part includes the choice tendency of patients' medical decision-making. The specific questionnaire design is shown in Appendix A.

The predictor variables consisted of a set of demographic and study variables that were selected based on the literature. The demographic variables (19) include patients' gender, age, education, religion, marital status, and the number of children, family annual income, and the main source of income. Study variables include the condition and severity of any disease (20, 21), the ratio of family monthly medical expenses to income (within 5 years) (22), and medical insurance status (23).

The outcome variable was the patients' medical decision-making choice tendency. According to the principle of optimization, the choice tendency of medical decision-making is set into four aspects, namely, treatment effect, treatment cost, treatment side effect, and treatment experience (8). Among them, the choice tendency of each category was sorted by scale (1 = very important, 2 = important, 3 = low importance, 4 = not important at all).

### 2.2. Methods

Due to the small sample size (*n* = 248), insufficient data exist at each level of the medical decision-making tendency, which will affect the performance of the model. So the medical decision-making choice tendency can be further divided into two intervals: important and unimportant (which is 1 or 2 = important, 3 or 4 = unimportant).

The traditional ML algorithm model has higher generalization ability than the deep learning algorithm due to the limited sample size (24, 25). Furthermore, considering that the samples in this study are structured data, the predicted medical decision-making tendency of patients is a binary classification problem. As a result, the binary classification algorithm in supervised ML is used in this study (26). Currently, binary classification algorithms can be divided into single classification algorithms and ensemble algorithms with good performance, among which single algorithm mainly includes Naive Bayes classification, k-nearest neighbor (KNN) classification, decision tree (DT) classification, support vector machine (SVM) classification, and ensemble algorithms including Bagging classification, Random Forest classification, and Boosting series (27). The ensemble classification algorithm is more suitable for complex data, but the prediction speed is significantly reduced compared with the single algorithm. Additionally, the premise of the Naive Bayes algorithm is that it must conform to the independence attribute of samples (28). Thus, taking into account the data characteristics and prediction problems of the samples in this study, KNN classification, DT classification, and SVM classification are chosen to predict the patient's medical decision-making tendency, and the performance of the three classification algorithms is compared and analyzed.

The classification algorithm's performance is then evaluated using the following evaluation indexes: Accuracy rate, Precision rate, Recall rate, and F1-Score (29).


(1) $$\begin{array}{l} \text{Accuracy }=\frac{TP+TN}{TP+FP+TN+FN} \end{array}$$

**Figure 1:**
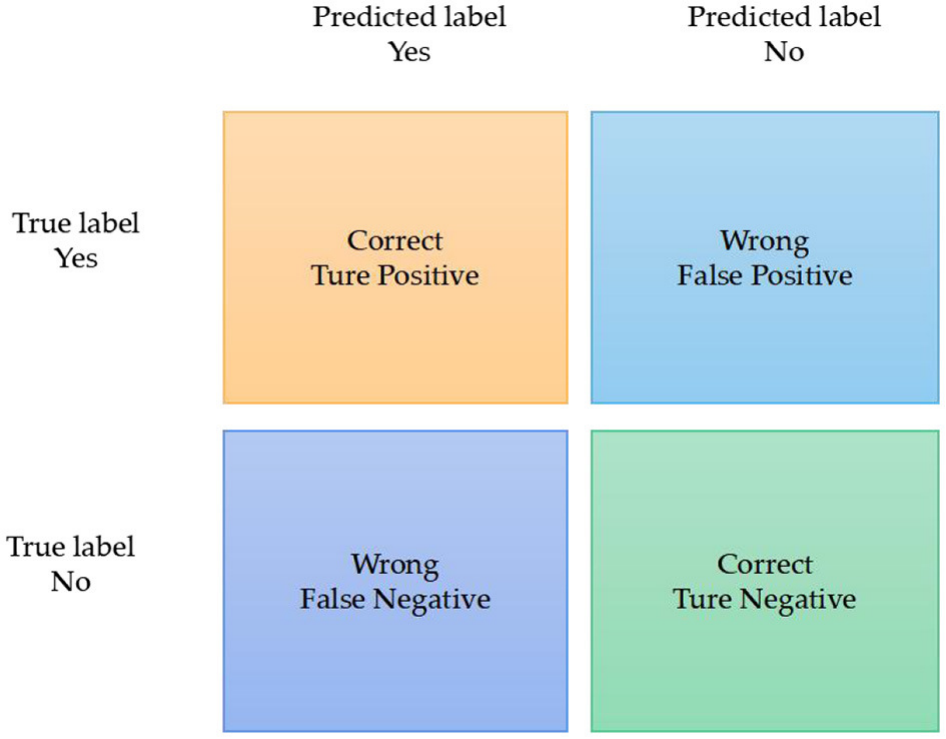
Confusion matrix diagram.

For binary problems, the prediction results are classified into positive or negative categories. As shown in Figure 1, the True Positive (*TP*) refers to the number of positive classes predicted into positive; True Negative (*TN*) refers to the number of negative classes predicted into negative; False Positive (*FP*) refers to the number of positive classes predicted into negative; False Negative (*FN*), Refers to the number of negative classes predicted to be positive. Therefore, the Accuracy rate can be seen as in Equation (1), which represents the accuracy rate of the predicted quantity and measures the ability of the model to avoid errors.


(2) $$\begin{array}{l} \text{Precision }=\;\frac{TP}{TP+FP} \end{array}$$

The Precision rate represents the ability of the model to predict positive samples, as shown in Equation (2).


(3) $$\begin{array}{l} \text{Recall}=\frac{TP}{TP+FN} \end{array}$$

The Recall rate represents the ability of positive samples to be recognized, as shown in Equation (3).


(4) $$\begin{array}{l} \text{F}1-\text{Score}=\frac{2^{{}^{*}}P^{{}^{*}}R}{P+R}\; \end{array}$$

The F1-Score is the comprehensive evaluation index, F1-Score synthesizes the results of the Precision (*P*) rate and Recall (*R*) rate. When F1 is higher, the model is more effective, as shown in Equation (4).

## 3. Results and discussion

Following the global crisis triggered by the COVID-19 pandemic, the world economy has plunged into a slump, with a sharp increase in instability and uncertainty. At the same time, medical resources are strained, and the conflict between physicians and patients has intensified. Up to 18,670 medical dispute cases were recorded by Chinese courts in 2020; an increase of 2.98% compared with 2019 (30). During the COVID-19 pandemic, both physicians and patients in clinical settings are in dire need of decision support systems in their shared decision-making process. With the increase of medical and health data, predictive tools based on artificial intelligence technology can effectively guide overwhelmed medical staff to make informed decisions in the complex clinical diagnosis and treatment environment (31). In this context, the research conducted this prediction study on patients' choice tendency for medical decision-making based on ML algorithms. The research results will help to build a patient-centered medical decision-making assistance system to assist medical staff in quickly understanding the patient's decision-making tendency, effectively resolve the conflict of interest between physicians and patients, and ultimately help physicians and patients to achieve scientific decision-making. Combined with the empirical results of this study, this paper discusses the development prospects of the patient-centered medical decision assistance system from the following three aspects.

### 3.1. Patient-centered medical decision-making assistance systems help to resolve conflicts of interest between physicians and patients

The key point of predicting patients' medical decision-making choice tendency is to select effective predictor variables. However, the influencing factors affecting decision-making are complex and the data is difficult to collect. According to the above, 12 predictor variables were selected for feature processing according to the above, and the medical decision-making preference was classified into four aspects: treatment effect; treatment cost; treatment side effect; and treatment experience.

The descriptive analysis of specific variables is shown in Table 1. The number of male patients was similar to that of female patients, with 46% males and 54% females. Patients aged 18–35 years had the highest proportion (53%), and the proportion of bachelor's degree or above was the highest, at 46%. Among the surveyed patients, 52% thought the treatment effect was very important, 36% thought the treatment cost was very important, 33% thought the treatment side effect was very important, and 60% thought the treatment experience was very important. Based on the descriptive statistical analysis of variables, linear regression analysis was performed with basic information about patients as independent variables and the four types of medical choice tendencies as dependent variables. The specific analysis results are shown in Table 2, in which the regression results of treatment effect can be seen in model 1; treatment costs, model 2; treatment side effects, model 3; and treatment experience, model 4.

**Table 1:** Descriptive statistics of variables.

**Variable name**	**Option name**	**Frequency (*n*)**	**Percentage (%)**
Gender	Male	114	46
	Female	134	54
Age (years)	18–35	131	53
	36–60	63	25
	61–79	42	17
	More than 80	12	5
Education	Junior high school and below	65	26
	Senior high school (technical secondary school) and junior college	69	28
	Bachelor degree or above	114	46
Condition of the disease	Serious illness	43	17
	Non-serious illness	205	83
Severity of the disease	Critical	11	4
	High	25	10
	Average	113	46
	Moderate	38	15
	Low	61	25
Family's annual income	Below $4,422	27	11
	$4,422–$7,370	24	10
	$7,370–$29,480	145	58
	$29,480–$73,700	41	17
	Over $73,700	11	4
Main source of income	Wage income	205	83
	Self-employed	71	29
	Child support	34	14
	Parent support	14	6
	Subsistence allowances	9	4
	Other	44	18
Ratio of the family's monthly medical expenses to income (within 5 years)	<10%	152	61
	10–30%	63	25
	30–50%	12	5
	>50%	21	8
Medical insurance status	All at own expense	102	41
	Resident/employee basic medical insurance	143	58
	Free medical care	37	15
	Medical aid	3	1
	Commercial medical insurance and others	78	31
Religion	None	192	77
	Buddhism	29	12
	Christianity	13	5
	Islam	5	2
	Other	9	4
Marital status	Unmarried	69	28
	Married	176	71
	Divorced	1	0.4
	Widowed	0	0
	Other	2	0.8
Number of children	None	112	45
	1	84	34
	2	43	17
	3 or more	9	4
Medical decision-making tendency: Treatment effect	Very important	129	52
	Important	28	11
	Unimportant	86	35
	Very unimportant	5	2
Medical decision-making tendency: Treatment cost	Very important	90	36
	Important	1	0.4
	Unimportant	120	48
	Very unimportant	37	15
Medical decision-making tendency: Treatment side effect	Very important	81	33
	Important	1	0.4
	Unimportant	147	59
	Very unimportant	19	8
Medical decision-making tendency: treatment experience	Very important	150	60
	Important	17	7
	Unimportant	77	31
	Very unimportant	4	2

**Table 2:** Regression analysis of patients' choice tendency in medical decision-making.

**Variable**	**Dependent variable (medical decision-making tendency)**			
	**Model 1: Treatment effect**	**Model 2: Treatment cost**	**Model 3: Treatment side effect**	**Model 4: Treatment experience**
Gender (Female = 0)	0.091 (1.416)	−0.038 (−0.512)	−0.083 (−1.245)	0.030 (0.414)
Age	0.073 (0.986)	0.055 (0.822)	−0.106 (−1.746)	−0.022 (−0.333)
Education	−0.189^**^ (−2.865)	0.058 (0.862)	0.057 (0.926)	0.074 (1.133)
Condition of the disease	−0.151 (−1.436)	0.208 (1.621)	0.093 (0.796)	−0.179 (−1.503)
Severity of the disease	0.010 (0.032)	−0.115^**^ (−2.886)	−0.006 (−0.167)	0.117^**^ (3.112)
Family's annual income	−0.000 (−0.040)	0.002 (0.906)	−0.002 (−0.872)	−0.000 (−0.034)
Main source of income, Wage income (Other = 0)	0.171 (1.640)	−0.261^*^ (−2.347)	0.024 (0.235)	0.067 (0.669)
Main source of income, Self-employed (Other = 0)	0.034 (0.356)	−0.075 (−0.748)	0.021 (0.225)	0.021 (0.229)
Main source of income, Child support (Other = 0)	0.242 (1.862)	−0.341^*^ (−2.538)	0.126 (1.033)	−0.028 (−0.203)
Main source of income, Parent support (Other = 0)	−0.031 (−0.396)	0.041 (0.223)	0.080 (0.483)	−0.091 (−0.510)
Main source of income, Subsistence allowances (Other = 0)	0.221 (0.854)	−0.141 (−0.637)	−0.004 (−0.022)	−0.075 (−0.295)
Ratio of the family's monthly medical expenses to income (within 5 years)	0.050 (1.041)	−0.074 (−1.606)	0.028 (0.676)	−0.004 (−0.094)
Medical insurance status, All at own expense (Other = 0)	0.174 (1.727)	−0.351^**^ (−3.312)	0.232^*^ (2.375)	−0.055 (−0.549)
Medical insurance status, Resident/employee basic medical insurance (Other = 0)	−0.111 (−1.648)	0.097 (1.061)	0.158 (1.894)	−0.144 (−1.661)
Medical insurance status, Free medical care (Other = 0)	0.049 (0.044)	0.077 (0.627)	−0.029 (−0.258)	−0.097 (−0.984)
Medical insurance status, Medical aid (Other = 0)	−0.314 (−0.983)	0.651 (1.897)	−0.267 (−0.855)	−0.070 (−0.271)
Number of children	−0.041 (−0.803)	−0.056 (−1.025)	0.077 (1.554)	0.020 (0.399)
Marital status, Unmarried (Other = 0)	0.048 (0.038)	0.433 (1.047)	−0.028 (−0.075)	−0.453 (−1.159)
Marital status, Married (Other = 0)	0.375 (0.494)	0.220 (0.311)	−0.415 (−0.645)	−0.180 (−0.273)
Marital status, Divorced (Other = 0)	0.044 (0.056)	0.340 (0.792)	−0.064 (−0.163)	−0.321 (−0.795)
Religion, Islam (None = 0)	−0.223 (−0.856)	−0.532 (−1.632)	0.539 (1.817)	0.216 (0.742)
Religion, Christianity (None = 0)	−0.203 (−0.946)	−0.363 (−1.363)	0.562 (2.317)	0.003 (0.051)
Religion, Buddhism (None = 0)	−0.387 (−2.066)	−0.313 (−1.423)	0.425 (2.120)	0.275 (1.393)
Religion, Other (None = 0)	−0.176 (−0.975)	−0.138 (−0.694)	−0.444^*^ (−2.460)	−0.130 (−0.694)
Constant	2.766^**^ (4.865)	2.723^**^ (4.413)	1.977^**^ (3.517)	2.534^***^ (4.451)
*N*	248	248	248	248
*R^2^*	0.333	0.234	0.114	0.114

(1) The brackets of the variables are the reference group; (2) The numbers in parentheses are the *t*-values; (3) ^***^, ^**^, and ^*^ are significance levels of 1%, 5%, and 10%, respectively.

In medical services, physicians commit to practice ethically and to putting patient wellbeing first (32). As a result, in addition to the diagnosis and treatment of patient diseases, they also need to pay attention to other aspects of patient medical needs. First, for patients who pay attention to the treatment effect, the regression analysis results in Table 2 show that the order of the educational level of this type of patients and the importance of the treatment effect (1 = very important, 4 = very unimportant) has a negative effect at the 5% significance level. That is, the higher the educational level of the patient, the more attention is paid to the effect of treatment (33, 34). This finding also suggests that patients with higher education levels have higher levels of health literacy (35), and are more concerned and aware of disease treatment options. In the process of providing medical services to this type of patient, medical staff can give a more professional introduction based on the patient's knowledge background, increase the explanation of the theoretical knowledge of the disease, and enrich the patient's understanding of their disease, thereby helping the patient to improve the treatment effect. Second, for patients who pay attention to treatment costs, according to the regression analysis results in Table 2, it can be seen that the medical insurance status of this type of patient is fully self-paid, which has a negative effect on the importance of treatment costs at the 5% significance level. In other words, patients who fully self-pay are more aware of the cost of treatment than patients with other conditions. As a result, in the process of providing medical services for this type of patient, medical staff should pay more attention to the cost of treatment, and can provide patients with alternative cost-effective treatment options (36), such as the use of domestic drugs in the process of treatment. Third, Table 2 shows that patients with religious beliefs in this group are more likely to pay attention to treatment side effects than those without religious beliefs. Religion may play a role here, specifically the religious taboos of China's ethnic minorities (37), which hold that women should not compromise their bodies in any way and thus reject surgical treatment options like organ removal. It's clear that physicians and nurses planning care for patients with religious restrictions need to pay special attention to the possibility of treatment-related harm. Fourth, for patients who pay attention to treatment experience, it can be seen that the patient's disease status and the ranking of important procedures for treatment experience have a positive effect at the 5% significance level. This demonstrates that patients with severe diseases are more concerned about the pain that treatment may bring (38). As a result, medical personnel should strengthen the level of nursing care for these patients with serious illnesses, reduce the pain of patient treatment, and enhance their treatment experience.

The establishment of a patient-centered medical decision-making assistance system will help medical staff to understand different types of patients and provide patients with targeted and humanized medical services. This will assist in resolving any conflicts of interest between physicians and patients, thereby reducing the cost of communication between them and improving the quality of medical services.

### 3.2. Machine learning technology effectively promotes the development of patient-centered medical decision-making assistance systems

Following the regression analysis of patient medical decision choice tendency, the ML algorithm was used to predict patient choice tendency. The ML algorithm is implemented in this study using the sklearn tool in Python programming language. The One-Hot encoding of categorical variables was carried out before the operation of the model, and the parameters were adjusted during the construction of the model.

**Figure 2:**
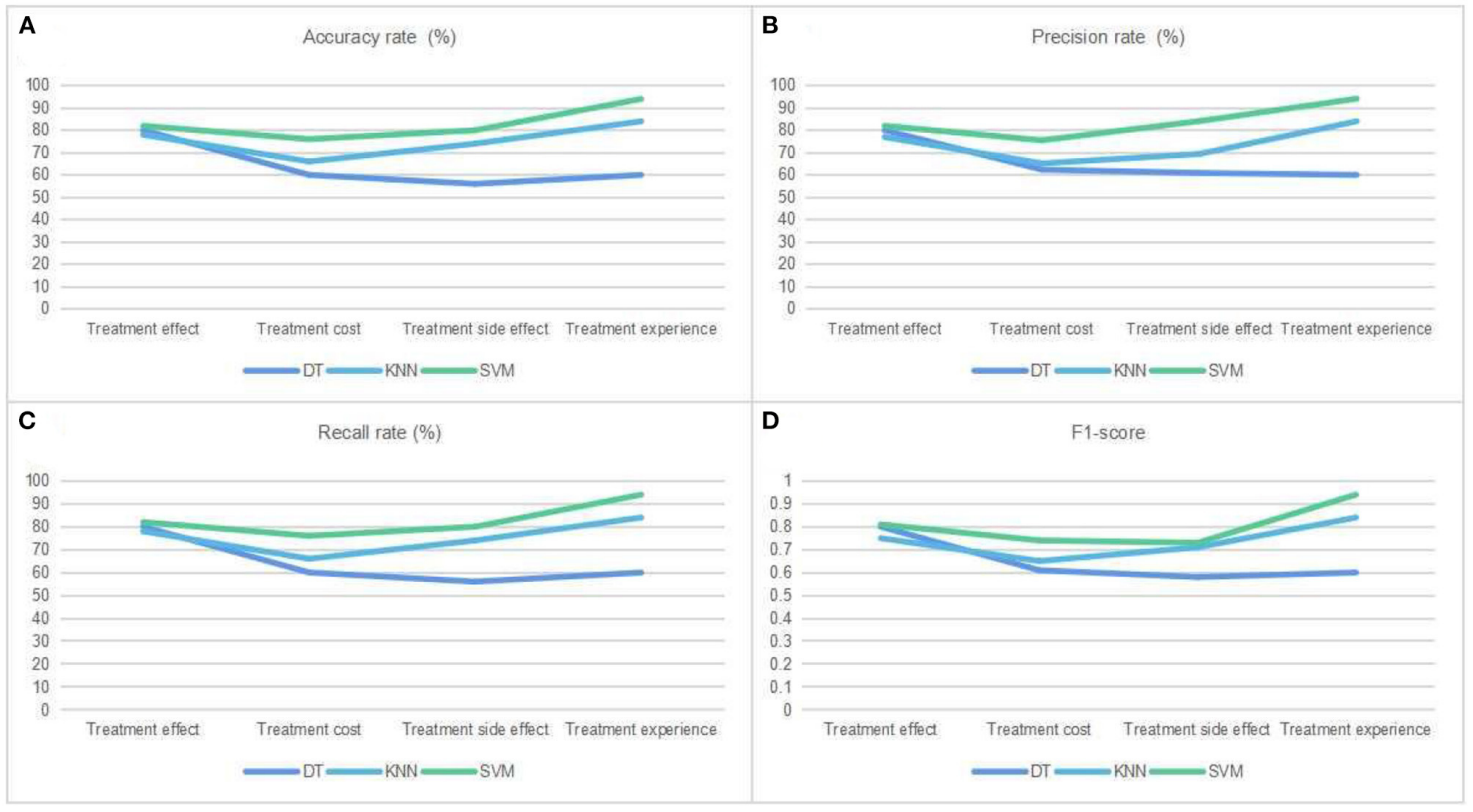
Performance comparison of three kinds of classification algorithms. **(A)** The accuracy rate of patients' medical decision selection tendency was predicted by three ML classification algorithms: decision tree (DT), k-nearest neighbor (KNN), and support vector machine (SVM). **(B)** Same as **(A)**, but for precision rate. **(C)** Same as **(A)**, but for recall rate. **(D)** Same as **(A)**, but for F1-score.

In order to compare the performance of the three algorithms, the accuracy rate, precision rate, recall rate, and F1-Score of the evaluation indexes are selected. The specific results of the performance evaluation indexes can be seen in Tables 3–5. Accuracy for treatment effect, cost, side effect, and patient experience, respectively, is 80%, 60%, 56%, and 60% when the DT algorithm is used to predict patient preferences, compared to 78%, 66%, 74%, and 84% when the KNN algorithm is used, and 82%, 76%, 80%, and 94% when the SVM algorithm is used. It's clear that the SVM model has the highest accuracy rate overall. However, the accuracy rate alone is not enough to draw a conclusive conclusion about the model's performance. The F1-score is another metric that must be applied. According to the results, the F1-scores of the four types of choice tendencies predicted by the DT algorithm are 0.80, 0.61, 0.58, and 0.60; 0.75, 0.65, 0.71, and 0.84 for the KNN algorithm; and 0.81, 0.74, 0.73, and 0.94 for the SVM algorithm. Figure 2 depicts the specific performance comparison results.

**Table 3:** Model evaluation effect of decision tree algorithm.

**Categories of choice tendency in medical decision-making**	**Accuracy rate (%)**	**Precision rate (%)**	**Recall rate (%)**	**F1-score**
Treatment effect	80.00	80.00	80.00	0.80
Treatment cost	60.00	62.36	60.00	0.61
Treatment side effect	56.00	60.88	56.00	0.58
Treatment experience	60.00	60.00	60.00	0.60

The parameters of the decision tree algorithm are set as follows. The training set ratio is 0.8, the node split standard is “gini,” the node division mode is best, the minimum number of node split samples is 2, the minimum sample number of leaf nodes is 1, and the maximum tree depth is “No limit”.

**Table 4:** Model evaluation effect of k-nearest neighbors algorithm.

**Categories of choice tendency in medical decision-making**	**Accuracy rate (%)**	**Precision rate (%)**	**Recall rate (%)**	**F1-score**
Treatment effect	78.00	76.93	78.00	0.75
Treatment cost	66.00	65.14	66.00	0.65
Treatment side effect	74.00	69.38	74.00	0.71
Treatment experience	84.00	84.01	84.00	0.84

The parameters of the k-nearest neighbors are set as follows. The training set ratio is 0.8, the number of adjacent samples (*K*-value) is 5, the sample voting weight is “Equal voting rights” and the proximity search method is “auto”.

**Table 5:** Model evaluation effect of support vector machine algorithm.

**Categories of choice tendency in medical decision-making**	**Accuracy rate (%)**	**Precision rate (%)**	**Recall rate (%)**	**F1-score**
Treatment effect	82.00	81.98	82.00	0.81
Treatment cost	76.00	75.50	76.00	0.74
Treatment side effect	80.00	84.08	80.00	0.73
Treatment experience	94.00	94.11	94.00	0.94

The parameters of the support vector machine are set as follows. The training set ratio is 0.8, the error term penalty coefficient is 1.0, the kernel is rbf, the kernel coefficient values is 0.01, the multiclassification decision function is ovr, the model convergence parameter is 0.001, and the maximum number of iterations is 2,000.

In conclusion, the study's ML prediction results demonstrate that the SVM model has good performance, suggesting that the ML algorithm can help medical professionals gain a deeper understanding of patients' propensities for making certain choices. In the era of artificial intelligence, scholars propose to combine ML tools with decision-making to solve the impact of uncertain information on decision results (39). Some scholars even propose to use of ML algorithms to statistically analyze causal relationships and make predictions, thereby replacing human decision-making behavior (40). The involvement of ML algorithms in decision-making in the medical field is mature, such as in the diagnosis and prediction of diseases, including cancer, chronic kidney disease, Parkinson's disease, skin diseases, etc. (41).

Therefore, when taken together with the results of the prediction of patients' medical decision-making tendencies, it demonstrates that ML algorithms can effectively help medical staff quickly understand patients' needs (42), which in turn encourages the development of Patient-centered medical decision-making assistance systems (43). This system can enhance the trust between physicians and patients (44). Ultimately helps them reach a shared decision-making model (45) and the attainment of scientifically grounded choice.

### 3.3. Opportunities and challenges of building a patient-centered medical decision-making assistance system under the background of artificial intelligence

The healthcare system has been overwhelmed by the Covid-19 pandemic (46), and mental health issues are on the rise among healthcare workers (47). The likelihood of harmful medical disputes is bound to rise in such a setting. In this context, there is a huge opportunity to build a patient-centered medical decision aid system based on artificial intelligence technologies such as ML algorithms. The patient-centered medical decision assistance system is helpful to strengthen the understanding between physicians and patients, resolve conflicts of interest and assist in realizing scientific decision-making, which has important application value in clinical practice.

However, the following obstacles will also need to be overcome during the construction of the auxiliary decision-making system. First, in the process of system development, it is necessary to avoid security issues such as patient medical information data leakage. Due to the enormous value of data in the medical field, it is frequently the target of theft by criminal groups who profit from the malicious use of data (48). Medical data leakage will aggravate patient distrust of medical institutions, which will lead to major crises in medical institutions. Therefore, in the development of auxiliary decision-making systems, it is necessary to strengthen the protection of patient privacy and rights through information technology (49). Second, in the operation process of the auxiliary decision-making system, patients are required to provide not only disease information, but also other demographic information, which will increase the medical burden of patients, such as elderly patients with low information literacy levels (50). According to relevant studies (51, 52), only 16.7% of elderly patients in western Chinese cities meet the health literacy level. In order to reduce patients' confusion and concern, medical staff must patiently explain and guide them while gathering information for such patients. Third, in the application process of the auxiliary decision-making system, it is also necessary to provide corresponding training in information technology knowledge to enhance the information literacy of medical staff. According to studies, health information literacy is becoming increasingly important in both developed and developing countries (53). As a result, information skills training for medical staff is very important, which will help to improve the interpretation ability of data decision-making results, and then improve the quality of medical decision-making.

## 4. Conclusions

Medical decision assistance systems are the growing trend that helps physicians and patients make scientific decisions and resolve conflicts of interest. The purpose of this research was to suggest a method for using the ML algorithm to predict a patient's preferences when making important medical decisions. The findings demonstrate the SVM algorithm's strong predictive abilities, which suggests that algorithms such as ML in artificial intelligence technology can contribute to the development and application of patient-centered medical decision assistance systems.

Furthermore, this study still has some limitations. First, due to conditional constraints, the sample size of the questionnaire survey on patients' medical decision-making preferences is insufficient, resulting in the inability of some algorithms to achieve good performance. Second, the selection of features for this study through literature review, which may cause noise in some features and affect the generalization ability of the model. It is hoped that in future research work, the sample size can be further expanded and the method of feature selection can be optimized, which will then be compared with ensemble learning or deep learning algorithms to improve the prediction model's performance and generalization ability.

## Data availability statement

The raw data supporting the conclusions of this article will be made available by the authors, without undue reservation.

## Ethics statement

Ethical review and approval was not required for the study of human participants in accordance with the local legislation and institutional requirements. Written informed consent from the patients/participants was not required to participate in this study in accordance with the national legislation and the institutional requirements.

## Author contributions

YL and JL: conceptualization and validation. YL and ZY: methodology, software, data curation, and visualization. YL and QX: formal analysis. JL: investigation, writing-review and editing, supervision, project administration, and funding acquisition.

## References

[1] Javed AR Fahad LG Farhan AA Abbas S Srivastava G Parizi RM. Automated cognitive health assessment in smart homes using machine learning. Sustain Cities Soc. (2021) 65:102572. 10.1016/j.scs.2020.102572

[2] Chamoun N Matta S Aderian SS Salibi R Salameh P Tayeh G. A prospective observational cohort of clinical outcomes in medical inpatients prescribed pharmacological thromboprophylaxis using different clinical risk assessment models (COMPT RAMs). Sci Rep. (2019) 9:18366. 10.1038/s41598-019-54842-3 31797897 PMC6892868

[3] Hill C Knafl KA Santacroce SJ. Family-centered care from the perspective of parents of children cared for in a pediatric intensive care unit: an integrative review. J Pediatr Nurs. (2018) 41:22–33. 10.1016/j.pedn.2017.11.007 29153934 PMC5955783

[4] Xiao L Peng M Liu Y Zhang L. Analysis of the conceptual framework of patient participation in medical decision making. Med Philos. (2021) 42:20–5.

[5] Yuan J. Involve patients in their own medical decisions. Health Manage. (2014) 3:58–59.

[6] Schoenfeld EM Kanzaria HK Quigley DD Marie PS Nayyar N Sabbagh SH. Patient preferences regarding shared decision making in the emergency department: findings from a multisite survey. Acad Emerg Med. (2018) 25:1118–28. 10.1111/acem.13499 29897639 PMC6185792

[7] Zhou J Li K. The importance and ways of patient participation in medical decision-making. Chin Rur Health Serv Admin. (2012) 32:611–12.

[8] Liu J Yan J. Medical Ethics. Wuhan: Huazhong University of Science and Technology Press. (2019).

[9] Kumar KA Singh Y Sanyal S. Hybrid approach using case-based reasoning and rule-based reasoning for domain independent clinical decision support in ICU. Expert Syst Appl. (2009) 36:65–71. 10.1016/j.eswa.2007.09.054

[10] Zhou X Li Y Liang W. CNN-RNN based intelligent recommendation for online medical pre-diagnosis support. IEEE ACM Trans Comput Biol Bioinform. (2020) 18:912–21. 10.1109/TCBB.2020.2994780 32750846

[11] Sun H Liu Z Wang G Lian W Ma J. Intelligent analysis of medical big data based on deep learning. IEEE Access. (2019) 7:142022–37. 10.1109/ACCESS.2019.2942937

[12] Pourhomayoun M Shakibi M. Predicting mortality risk in patients with COVID-19 using machine learning to help medical decision-making. Smart Health. (2021) 20:100178. 10.1016/j.smhl.2020.100178 33521226 PMC7832156

[13] Salah HT Muhsen IN Salama ME Owaidah T Hashmi SK. Machine learning applications in the diagnosis of leukemia: current trends and future directions. Int J Lab Hematol. (2019) 41:717–25. 10.1111/ijlh.13089 31498973

[14] Vorwerk C Loryman B Coats TJ Stephenson JA Gray LD Reddy G. Prediction of mortality in adult emergency department patients with sepsis. Emerg Med J. (2009) 26:254–58. 10.1136/emj.2007.053298 19307384

[15] Valdes G Simone II CB Chen J Lin A Yom SS Pattison AJ. Clinical decision support of radiotherapy treatment planning: a data-driven machine learning strategy for patient-specific dosimetric decision making. Radiother Oncol. (2017) 125:392–97. 10.1016/j.radonc.2017.10.014 29162279

[16] Christo VRE Nehemiah HK Nahato KB Brighty J Kannan A. Computer assisted medical decision-making system using genetic algorithm and extreme learning machine for diagnosing allergic rhinitis. Int J Bioinspir Comput. (2020) 16:148–57. 10.1504/IJBIC.2020.111279

[17] Cai CJ Reif E Hegde N Hipp J Kim B Smilkov D. Human-centered tools for coping with imperfect algorithms during medical decision-making. Paper Presented at the Proceedings of the 2019 Chi Conference on Human Factors in Computing Systems. Glasgow (2019). 10.1145/3290605.3300234

[18] London AJ. Artificial intelligence and black-box medical decisions: accuracy versus explainability. Hastings Cent Rep. (2019) 49:15–21. 10.1002/hast.973 30790315

[19] Thompson SC Pitts JS Schwankovsky L. Preferences for involvement in medical decision-making: situational and demographic influences. Patient Educ Couns. (1993) 22:133–40. 10.1016/0738-3991(93)90093-C 8153035

[20] Degner LF Sloan JA. Decision making during serious illness: what role do patients really want to play? J Clin Epidemiol. (1992) 45:941–50. 10.1016/0895-4356(92)90110-9 1432023

[21] Hunink MGM Weinstein MC Wittenberg E Drummond MF Pliskin JS Wong JB. Decision Making in Health and Medicine: Integrating Evidence and Values. Cambridge University Press (2014). 10.1017/CBO9781139506779

[22] Youm J Chan V Belkora J Bozic KJ. Impact of socioeconomic factors on informed decision making and treatment choice in patients with hip and knee OA. J Arthroplasty. (2015) 30:171–75. 10.1016/j.arth.2014.09.006 25301018

[23] Murray E Pollack L White M Lo B. Clinical decision-making: patients' preferences and experiences. Patient Educ Couns. (2007) 65:189–96. 10.1016/j.pec.2006.07.007 16956742

[24] Zhang Y Ling C. A strategy to apply machine learning to small datasets in materials science. Npj Comput Mater. (2018) 4:25. 10.1038/s41524-018-0081-z35478886

[25] Janiesch C Zschech P Heinrich K. Machine learning and deep learning. Electr Mark. (2021) 31:685–95. 10.1007/s12525-021-00475-2

[26] Zhou Z-H. Machine Learning. Singapore: Springer Nature. (2021). 10.1007/978-981-15-1967-3

[27] Yang J Peirui Q Yongmei L Ning W. A review of machine learning classification problems and algorithms. Stat Decis. (2019) 35:36–40.

[28] Webb GI Keogh E Miikkulainen R. Naïve Bayes. In: Encyclopedia of Machine Learning. Sammut C Webb GI , editor. Boston, MA: Springer (2010) 713–14. 10.1007/978-0-387-30164-8_576

[29] Jiao Y Du P. Performance measures in evaluating machine learning based bioinformatics predictors for classifications. Quant Biol. (2016) 4:320–30. 10.1007/s40484-016-0081-2

[30] Yifahui. Big Data Report on China's Medical Damage Liability Dispute Cases in 2020. Beijing: YIFAHUI Medical Lawyer Network (2020).

[31] Debnath S Barnaby DP Coppa K Makhnevich A Kim EJ Chatterjee S. Machine learning to assist clinical decision-making during the COVID-19 pandemic. Bioelectron Med. (2020) 6:1–8. 10.1186/s42234-020-00050-8 32665967 PMC7347420

[32] Nie J-B Cheng Y Zou X Gong N Tucker JD Wong B. The vicious circle of patient–physician mistrust in China: health professionals' perspectives, institutional conflict of interest, and building trust through medical professionalism. Dev World Bioeth. (2018) 18:26–36. 10.1111/dewb.12170 28922547

[33] Kane CJ Lubeck DP Knight SJ Spitalny M Downs TM Grossfeld GD. Impact of patient educational level on treatment for patients with prostate cancer: data from CaPSURE. Urology. (2003) 62:1035–39. 10.1016/S0090-4295(03)00778-7 14665350

[34] Haider SI Johnell K Weitoft GR Thorslund M Fastbom J. The influence of educational level on polypharmacy and inappropriate drug use: a register-based study of more than 600,000 older people. J Am Geriatr Soc. (2009) 57:62–9. 10.1111/j.1532-5415.2008.02040.x 19054196

[35] Connor M Mantwill S Schulz PJ. Functional health literacy in Switzerland—validation of a German, Italian, and French health literacy test. Patient Educ Couns. (2013) 90:12–7. 10.1016/j.pec.2012.08.018 23089240

[36] Neumann PJ Sandberg EA Bell CM Stone PW Chapman RH. Are pharmaceuticals cost-effective? A review of the evidence: do drug treatments give value for the money? Careful analysis can yield useful information, this study finds. Health Aff. (2000) 19:92–109. 10.1377/hlthaff.19.2.92 10718025

[37] Bai X. Research on the Taboo Culture of Yi People. Doctorate, Sichuan University (2001).

[38] Kemp HI Laycock H Costello A Brett SJ. Chronic pain in critical care survivors: a narrative review. Br J Anaesth. (2019) 123:e372–84. 10.1016/j.bja.2019.03.025 31126622 PMC6676238

[39] Tulabandhula T Rudin C. On combining machine learning with decision making. Mach Learn. (2014) 97:33–64. 10.1007/s10994-014-5459-7

[40] Balasubramanian N Ye Y Xu M. Substituting human decision-making with machine learning: implications for organizational learning. Acad Manage Rev. (2022) 47:448–65. 10.5465/amr.2019.0470

[41] Jayatilake SMDAC Ganegoda GU. Involvement of machine learning tools in healthcare decision making. J Healthc Eng. (2021) 2021:6679512. 10.1155/2021/6679512 33575021 PMC7857908

[42] Lin C-C Hwang S-J. Patient-centered self-management in patients with chronic kidney disease: challenges and implications. Int J Environ Res Public Health. (2020) 17:9443. 10.3390/ijerph17249443 33339300 PMC7766278

[43] van der Eijk M Faber MJ Al Shamma S Munneke M Bloem BR. Moving towards patient-centered healthcare for patients with Parkinson's disease. Parkinsonism Relat Disord. (2011) 17:360–64. 10.1016/j.parkreldis.2011.02.012 21396874

[44] Braddock CH III Snyder L Neubauer RL Fischer GS American American College of Physicians Ethics Professionalism and Human Rights Committee The The Society of General Internal Medicine Ethics Committee*. The patient-centered medical home: an ethical analysis of principles and practice. J Gen Intern Med. (2013) 28:141–46. 10.1007/s11606-012-2170-x 22829295 PMC3539020

[45] Col NF Solomon AJ Springmann V Garbin CP Ionete C Pbert L. Whose preferences matter? A patient-centered approach for eliciting treatment goals. Med Decis Making. (2018) 38:44–55. 10.1177/0272989X17724434 28806143 PMC5929460

[46] Salman AM Ahmed I Mohd MH Jamiluddin MS Dheyab MA. Scenario analysis of COVID-19 transmission dynamics in Malaysia with the possibility of reinfection and limited medical resources scenarios. Comput Biol Med. (2021) 133:104372. 10.1016/j.compbiomed.2021.104372 33864970 PMC8024227

[47] De Kock JH Latham HA Leslie SJ Grindle M Munoz S-A Ellis L. A rapid review of the impact of COVID-19 on the mental health of healthcare workers: implications for supporting psychological well-being. BMC Public Health. (2021) 21:1–18. 10.1186/s12889-020-10070-3 33422039 PMC7794640

[48] Kinoon MA Omar M Mohaisen M Mohaisen D. Security breaches in the healthcare domain: a spatiotemporal analysis. Paper Presented at the International Conference on Computational Data and Social Networks. Cham: Springer (2021). 10.1007/978-3-030-91434-9_16

[49] Hoens TR Blanton M Steele A Chawla NV. Reliable medical recommendation systems with patient privacy. ACM Trans Intell Syst Technol. (2013) 4:1–31. 10.1145/2508037.2508048

[50] Olphert W Damodaran L. Older people and digital disengagement: a fourth digital divide? Gerontology. (2013) 59:564–70. 10.1159/000353630 23969758

[51] Javed AR Shahzad F ur Rehman S Zikria YB Razzak I Jalil Z. Future smart cities requirements, emerging technologies, applications, challenges, and future aspects. Cities. (2022) 129:103794. 10.1016/j.cities.2022.10379434540568

[52] Chengbo L Guo Y. The effect of socio-economic status on health information literacy among urban older adults: evidence from Western China. Int J Environ Res Public Health. (2021) 18:3501. 10.3390/ijerph18073501 33800562 PMC8036692

[53] Ottosen T Mani NS Fratta MN. Health information literacy awareness and capacity building: present and future. IFLA J. (2019) 45:207–15. 10.1177/0340035219857441

