# Supplementary material for: Prediction of patient choice tendency in medical decision-making based on machine learning algorithm
Source: Front Public Health. 2023 Feb 24;11:1087358. doi: 10.3389/fpubh.2023.1087358 (PMC9998498; doi:10.3389/fpubh.2023.1087358)
Supplement: Supplementary file 1 [file Data_Sheet_1.pdf]

Appendix A

Questionnaire on patients' medical decision-making tendency based on optimization principle

Dear Sir/Madam,

In order to understand what are your main medical decision-making tendency in the treatment process? How do you feel about the importance of four types of choice tendencies? Understanding these issues can help strengthen doctor-patient communication, and allow medical staff to help you formulate a more reasonable medical plan, thereby improving the effectiveness of diagnosis and treatment.

We promise that there is no commercial interest in this survey, and will keep your personally identifiable information confidential during the data processing. Participation in this survey is entirely voluntary, and you may decline to accept it. Thank you very much for your strong support for our survey!

1. Basic Information Survey (The following questions are required.)

1.1. What's your gender? (Single choice)

○ Male

○ Female

1.2. What's your age (years)? (Single choice)

○ 18~35

○ 36-60

○ 61-79

○ Over 80

1.3. What is your educational background?(Single choice)

○ Junior high school and below

○ Senior high school (technical secondary school) and junior college

○ Bachelor degree or above

1.4. What is the condition of your illness? (Single choice)

○ Serious illness

○ Non serious illness

1.5. How do you feel about the severity of your illness right now? (Single choice)

○ Critical

○ High

○ Average

○ Moderate

○ Low

1.6. How much is your family's annual income? (Fill in the Blank question])

____

1.7. What is your main source of income? (Multiple choice)

□ Wage income

□ Self-employed

□ Child support

□ Parent support

□ Subsistence allowances

□ Other

1.8. In the past five years, what percentage of your family's average monthly medical expenses (RMB) to total income? (Single choice)

○ Below 10%

○ 10%-30%

○ 30%-50%

○ Above 50%

1.9. What type of medical insurance do you have? (Multiple choice)

□ All at own expense

□ Resident/employee basic medical insurance

□ Free medical care

□ Medical aid

□ Commercial medical insurance and others

1.10. What is your religion? (Single choice)

○ None

○ Buddhism

○ Christianity

○ Islam

○ Other

1.11. What is your marital status?

○ Unmarried

○ Married

○ Divorced

○ Widowed

○ Other

1.12. How many children do you have?

○ None

○ 1

○ 2

○ 3 or more

2. Investigation of medical decision-making choice tendency.

When the medical staff formulates the medical plan, please rank the importance of the four factors of treatment effect, treatment cost, treatment side effects, and treatment experience according to your own family situation, disease status and other factors (1=Very Important, 2=Important, 3=Low Importance, 4=Not At All Important).

2.1. Treatment effect

○ Very Important

○ Important

○ Unimportant

○ Very Unimportant

2.2. Treatment cost

○ Very Important

○ Important

○ Unimportant

○ Very Unimportant

2.3. Treatment side effects

○ Very Important

○ Important

○ Unimportant

○ Very Unimportant

2.4. Treatment experience

○ Very Important

○ Important

○ Unimportant

○ Very Unimportant
